# Potential for bias and low precision in molecular divergence time estimation of the Canopy of Life: an example from aquatic bird families

**DOI:** 10.3389/fgene.2015.00203

**Published:** 2015-06-08

**Authors:** Marcel van Tuinen, Christopher R. Torres

**Affiliations:** ^1^Department of Biology and Marine Biology, University of North Carolina at WilmingtonWilmington, NC, USA; ^2^Centre of Evolutionary and Ecological Studies, Marine Evolution and Conservation Group, University of GroningenGroningen, Netherlands; ^3^National Evolutionary Synthesis CenterDurham, NC, USA; ^4^Department of Integrative Biology, University of Texas at AustinAustin, TX, USA

**Keywords:** molecular clock, divergence time, calibration, fossil record, homoplasy, young clade, recent clade, shallow clade

## Abstract

Uncertainty in divergence time estimation is frequently studied from many angles but rarely from the perspective of phylogenetic node age. If appropriate molecular models and fossil priors are used, a multi-locus, partitioned analysis is expected to equally minimize error in accuracy and precision across all nodes of a given phylogeny. In contrast, if available models fail to completely account for rate heterogeneity, substitution saturation and incompleteness of the fossil record, uncertainty in divergence time estimation may increase with node age. While many studies have stressed this concern with regard to deep nodes in the Tree of Life, the inference that molecular divergence time estimation of shallow nodes is less sensitive to erroneous model choice has not been tested explicitly in a Bayesian framework. Because of available divergence time estimation methods that permit fossil priors across any phylogenetic node and the present increase in efficient, cheap collection of species-level genomic data, insight is needed into the performance of divergence time estimation of shallow (<10 MY) nodes. Here, we performed multiple sensitivity analyses in a multi-locus data set of aquatic birds with six fossil constraints. Comparison across divergence time analyses that varied taxon and locus sampling, number and position of fossil constraint and shape of prior distribution showed various insights. Deviation from node ages obtained from a reference analysis was generally highest for the shallowest nodes but determined more by temporal placement than number of fossil constraints. Calibration with only the shallowest nodes significantly underestimated the aquatic bird fossil record, indicating the presence of saturation. Although joint calibration with all six priors yielded ages most consistent with the fossil record, ages of shallow nodes were overestimated. This bias was found in both mtDNA and nDNA regions. Thus, divergence time estimation of shallow nodes may suffer from bias and low precision, even when appropriate fossil priors and best available substitution models are chosen. Much care must be taken to address the possible ramifications of substitution saturation across the entire Tree of Life.

## Introduction

Owing to the profound utility of molecular clock approaches in dating the tree of life, a concomitant increase is occurring in studies that investigate associated error, uncertainty or bias (e.g., van Tuinen and Hadly, 2004; Warnock et al., 2011; Parham et al., 2012; Dos Reis and Yang, 2013; Hipsley and Mueller, 2014). Many of these studies undoubtedly are stimulated by new computational approaches, but also by the ever-remaining temporal gap intimated in the fossil record across nearly all of life upon comparison with molecular timetrees. These studies have focused on both fossil calibration and molecular modeling and have contributed a wealth of fascinating insight and useful recommendations but also generated confounding trends.

Efforts in reconstructing the tree of life (Cracraft and Donoghue, 2004) and the corresponding timetree of life (Hedges and Kumar, 2009; Laurin, 2012) have, for good reason, emphasized the main branching order, yet, rapid acquisition of new data is now pushing timetree efforts into the canopy where divergence of the majority of living species has occurred. Such data may resolve the timing of recent radiations (including parallel adaptive radiations, e.g., Mahler et al., 2013); whether speciation events are synchronous or not (e.g., Smith et al., 2014); whether diversification events are synchronous or not (e.g., Armstrong et al., 2014); whether divergence corresponds to climate change (e.g., van Tuinen et al., 2004); and which population genetic and phylogeographic models provide greatest explanatory power (e.g., Arbogast et al., 2002). Therefore, it is fundamental that we understand the performance of divergence time estimation in such data sets. However, we lack insight into the performance of fossil-calibrated molecular data sets in divergence time estimation of young (<10 MY) nodes.

While much has been documented about uncertainty in divergence time estimation related to calibration choices (e.g., van Tuinen and Hedges, 2004; Marjanović and Laurin, 2007; Ho and Phillips, 2009; Parham et al., 2012), molecular substitution (e.g., Buckley et al., 2001; Arbogast et al., 2002) and clock model choice (e.g., Duchene et al., 2014; Ho and Duchene, 2014), most of these investigations have explicitly focused on nodes with higher taxonomic rank because they bracket a suitable fossil record or demonstrate a well-known evolutionary diversification event. Perhaps also contributing to the dearth of insight on the performance of time estimation of young clades, is that uncertainty in divergence time estimation is assumed to be less of an issue for recently evolved clades because the molecular clock is better approximated (Yang and Rannala, 2006; Brown and Yang, 2010, 2011), fossil record may be more complete (“pull of the recent”), rate variation is weaker (Rannala and Yang, 2007) and substitution saturation is less likely to significantly impact age estimation (Schwartz and Mueller, 2010; Lukoschek et al., 2012). It may also be envisioned that because of the commonly smaller number of nodes in clades with shorter divergence times, overall phylogenetic resolution may be more easily attained and computation constraints less of a hurdle in performing heuristic searches.

However, this view of younger clades being more readily “dateable” may paint an overly rosy picture. Indeed, many clades (including model adaptive radiations) have no or highly fragmentary fossil records making distant calibration the only avenue, whereas other clades with a well-understood early (stem) fossil record, may present choppy fossil evidence at the level of species divergence. Furthermore, the effect of incomplete lineage sorting of individual gene trees on divergence time estimation of a chosen species tree is not well studied from empirical data sets, and biases appear either to overestimate (Edwards and Beerli, 2000) or underestimate (DeGiorgio and Degnan, 2014) species divergence times. Because lineage sorting is a stochastic process, some intrinsic uncertainty in accuracy of divergence time estimation is expected. Although inclusion of multiple alleles per species is preferred in constructing species trees from gene trees, its impact on divergence time estimation too remains to be tested. Ignoring intraspecific heterozygosity leads to overestimation of interspecific divergences (Arbogast et al., 2002) but the significance of the absolute time bias at shallow temporal scale remains virtually untested (but see Lischer et al., 2014). Finally, many species-level studies include a fixed mutation rate (one calibration under a molecular clock), so the effect of calibration is not entirely known when a molecular clock is not held under a range of calibrations or where the distribution of polymorphic sites is not even across the tree (Lischer et al., 2014). When utilizing multiple calibrations, the distribution of individual priors has a profound effect on the joint prior (Warnock et al., 2011; Heath, 2012) and thus perhaps biases younger nodes differently than older nodes.

Because of the likelihood that bias and/or uncertainty in divergence time estimation are not equally distributed across the tree of life, it is valuable to investigate two possible scenarios: that uncertainty/bias increases with divergence time due to increasing molecular and fossil bias with time; and/or that uncertainty/bias is a function of temporal placement of calibrations, and thus context-specific. To further investigate the likelihood of each scenario, we used the avian taxon Phoenicopteridae (flamingos) as an example of a recently evolved clade. Flamingos are one of several aquatic families that contain a good fossil record, and recently (Torres et al., 2014) has been dated to be of young (<5 My) age. Among aquatic bird families, several fossil relatives fulfill best practice criteria for calibration (Ericson et al., 2006; Smith, 2010; Torres et al., 2014). We used multi-locus sampling in combination with various calibration approaches to test the relative effect of taxon, locus and calibration sampling on the inference of divergence time estimation of shallow vs. deep nodes. With this approach, we aimed to reject the null hypothesis that uncertainty/bias in divergence time estimation is independent of phylogenetic node age.

## Materials and Methods

### Sequence Choice and Calibration

Molecular methods for extraction, PCR and sequencing as well taxon sampling and locus sampling followed Torres et al. (2014), which included all six flamingo species, two grebe representatives, a penguin, tubenose and tropicbird, but with the new addition of six further outgroup taxa: *Fregata minor* (Fregatidae:frigatebirds), *Sula leucogaster* (Sulidae:gannets), *Phalacrocorax auritus* (Phalacrocoracidae:cormorants), *Anhinga melanogaster* (Anhingidae:anhingas), *Pelecanus occidentalis* (Pelecanidae:pelicans) and *Scopus umbretta* (Scopidae:hamerkop). This sampling of additional outgroup taxa satisfied the use of six vetted fossil constraints (see below). Locus sampling following Torres et al. (2014) included five intronic markers (NFKBIZ intron 6, Myoglobin intron 2, SLC29A4 intron 8, G3PDH intron 11, TIMM17A intron 3), 1 exon (ZENK exon 2), 1 3 UTR (ZENK), as well as two mtDNA markers (full Cytochrome b+barcode portion of Cytochrome Oxidase I).

Following the best-practice approach by Parham et al. (2012), we chose the following calibrations: crown Mirandornithes at 32.6 MY minimum (Torres et al., 2014), crown Podicipedidae at 8.7 MY minimum (Torres et al., 2014), stem Pelecanidae (*Pelecanus*–*Balaeniceps*/*Scopus* divergence) at 28.3 MY minimum (Louchart et al., 2011), Stem Anhingidae (*Anhinga*–*Phalacrocorax* divergence at 23.0 MY minimum (Mayr, 2001), Stem Sulidae (*Sula*-stem *Anhinga*) at 33.0 MY minimum (Mayr, 2002), stem Fregatidae (*Fregata*-stem Sulidae divergence) at 51.8 MY minimum (Olson, 1977), and stem Spheniscidae (*Spheniscus*–Procellariiformes divergence) at 60.5 MY minimum (Slack et al., 2006). For calibrations to meet the criteria of the best-practice approach, fossils must be linked to (1) specimen number, (2) apomorphy-based diagnosis in a published phylogenetic analysis-where its outcome is reconcilable with existing molecular phylogenies, (3) specific fossil locality and stratigraphic unit, and (4) reference to current geological age and details of numeric age selection (Parham et al., 2012).

### Reference Analysis

All divergence time analyses were run in Beast v1.8, convergence was checked, effect of burnin was tested and age estimates inferred in Tracer v1.6 with median age estimates reported here. Phylogenetic analyses were performed in Beast v1.8 (Bayesian) and MEGA v6.0 (Maximum Likelihood). Reference analysis to which all other sensitivity analyses were compared included the use of seven nDNA loci, complete species representation of Phoenicopteridae, inclusion of eight outgroup taxa, and six calibrations. The default prior age range distributions were lognormal with a standard deviation (SD) = 1 (thus adding an average of 10–15 MY to the minimum age as 95% values, in absence of suitable representative maximum ages); Posterior divergence time estimates obtained from the reference analysis were obtained after testing for proper convergence across triplicate runs of 40 M iterations. Additional sensitivity analyses (**Table 1**) were run in duplicate runs of 20 M iterations. In these additional analyses, several variables were altered one at a time from the reference analysis.

**Table 1:** Summary of 45 comparative MCMC divergence time runs, including justification and parameter settings.

MCMC run	#loci	#outgroups	#constraints	Comment
1	7	8	0	Testing joint prior
2	4	10	0	Testing joint prior
3	7	8	6	Reference run nDNA
4	8	8	6	See 3,+mtDNA
5	1	8	6	mtDNA only
6	7	8	6	See 3, 4 of 6 flamingo species excluded
7	4	10	6	See 3, 4 loci only^1^
8	4	10	7	See 7, anhinga constraint added
9	5	9	6	See 3, 5 loci only
10	5	9	7	See 9, anhinga constraint added
11	6	8	6	See 3, 6 loci only
12	4	10	2	See 7, youngest constraints only
13	5	9	2	See 9, youngest constraints only
14	6	8	2	See 11, youngest constraints only
15	7	8	2	See 3, youngest constraints only
16	4	3	2	See 12, reduced outgroup sampling^2^
17	5	3	2	See 13, reduced outgroup sampling^2^
18	6	3	2	See 14, reduced outgroup sampling^2^
19	7	3	2	See 15, reduced outgroup sampling^2^
20	6	8	6	See 3, 1 locus excluded
21	6	8	6	See 3, 1 locus excluded
22	6	8	6	See 3, 1 locus excluded
23	6	8	6	See 3, 1 locus excluded
24	6	8	6	See 3, 1 locus excluded
25	6	8	6	See 3, 1 locus excluded
26	7	8	6	See 3, constraint maxima increased to 65 or 30^3^
27	7	8	6	See 26, uniform prior distribution
28	7	8	6	See 26, 95% prior range at 65 or 30^3^
29	7	8	6	See 3, loci unpartitioned
30	7	8	7	See 3, + min_Phoenicopteridae_ = 5.33 constraint^4^
31	7	8	3	See 15, + min_Phoenicopteridae_ = 5.33 constraint^4^
32	7	8	4	See 3, sistergroup (grebe) taxa excluded
33	7	8	5	See 3, stem penguin constraint excluded
34	7	8	5	See 3, stem fregatebird constraint excluded
35	7	8	5	See 3, stem gannet constraint excluded
36	7	8	5	See 3, stem pelican constraint excluded
37	7	8	4	See 3, oldest constraints only
38	7	8	3	See 15, stem penguin constraint added
39	7	8	3	See 15, stem fregatebird constraint added
40	7	8	3	See 15, stem gannet constraint added
41	7	8	3	See 15, stem pelican constraint added
42	7	8	1	See 3, stem penguin constraint only
43	7	8	1	See 3,stem fregatebird constraint only
44	7	8	1	See 3,stem gannet constraint only
45	7	8	1	See 3,stem pelican constraint only

^1^Several combinations of loci were tested; analyses were limited to 4–7 loci (<4 loci failed to yield a robust phylogeny).

^2^Reduced outgroup sampling follows Torres et al. (2014).

^3^Prior range extended to 65 MY (K–T boundary) for all constraints, except youngest, Podicipedidae (30 MY; early Oligocene), constraint. Default lognormal prior distribution lognormal, SD = 1.

^4^Additional constraint is not based on fossil evidence, rather investigated to test consistency of Miocene/Pliocene divergence of crown Phoenicopteridae with overall aquatic bird.

### Locus Sensitivity Analyses

Several combinations of loci (each locus or region considered a separate partition) were tested to investigate the possible effect of a single locus driving posterior estimates or whether increased number of loci affected age uncertainty. mtDNA (full Cytochrome b+barcode portion of Cytochrome Oxidase I) was added as one locus to the nDNA data set to compare age estimates based on nDNA, mtDNA, or nDNA+mtDNA.

### Taxon Sensitivity Analyses

To test for effect of incomplete taxon sampling, we inferred whether including six (all) species or two species bracketing crown Phoenicopteridae yielded varying age estimates. We also ran an analysis where the sister group taxon (Podicipedidae) was excluded and several analyses with reduced outgroup representation. Differential coalescence rates of alleles among loci may contribute some variation across markers in divergence time estimation. These data too can be incorporated in multi-locus approaches by modeling of polymorphisms and incomplete lineage sorting such as in ^∗^BEAST (Heled and Drummond, 2010). This effect was not specifically tested in this paper but we refer to Torres et al. (2014) for discussion and analysis.

### Prior Distribution Sensitivity Analyses

Also, the effect of the shape of prior distributions was tested by altering prior age distributions from lognormal with default SD to normal and uniform distributions with SD > 1, effectively enlarging the soft maximum age, or by extending soft maximum ages according to absence of a fossil record for aquatic birds beyond particular geological boundaries (i.e., K–T boundary for oldest age of aquatic bird orders; Eocene–Oligocene boundary for Podicipedidae). Furthermore, different combinations of calibrations were tested to investigate the effect of number of calibrations, of temporal depth of calibrations (young vs. older calibrations), whether a specific calibration biased the joint prior more than others, or whether the nodal distance between (prior) calibration age and uncalibrated node posterior estimates influenced age uncertainty. ESS (Effective Sample Size) values of all parameters, calibration parameters, and age (prior) parameters were checked at different iteration intervals and across duplicate runs to investigate possible difference in convergence of shallow versus deeper nodes.

## Results

### Phylogeny

**FIGURE 1:**
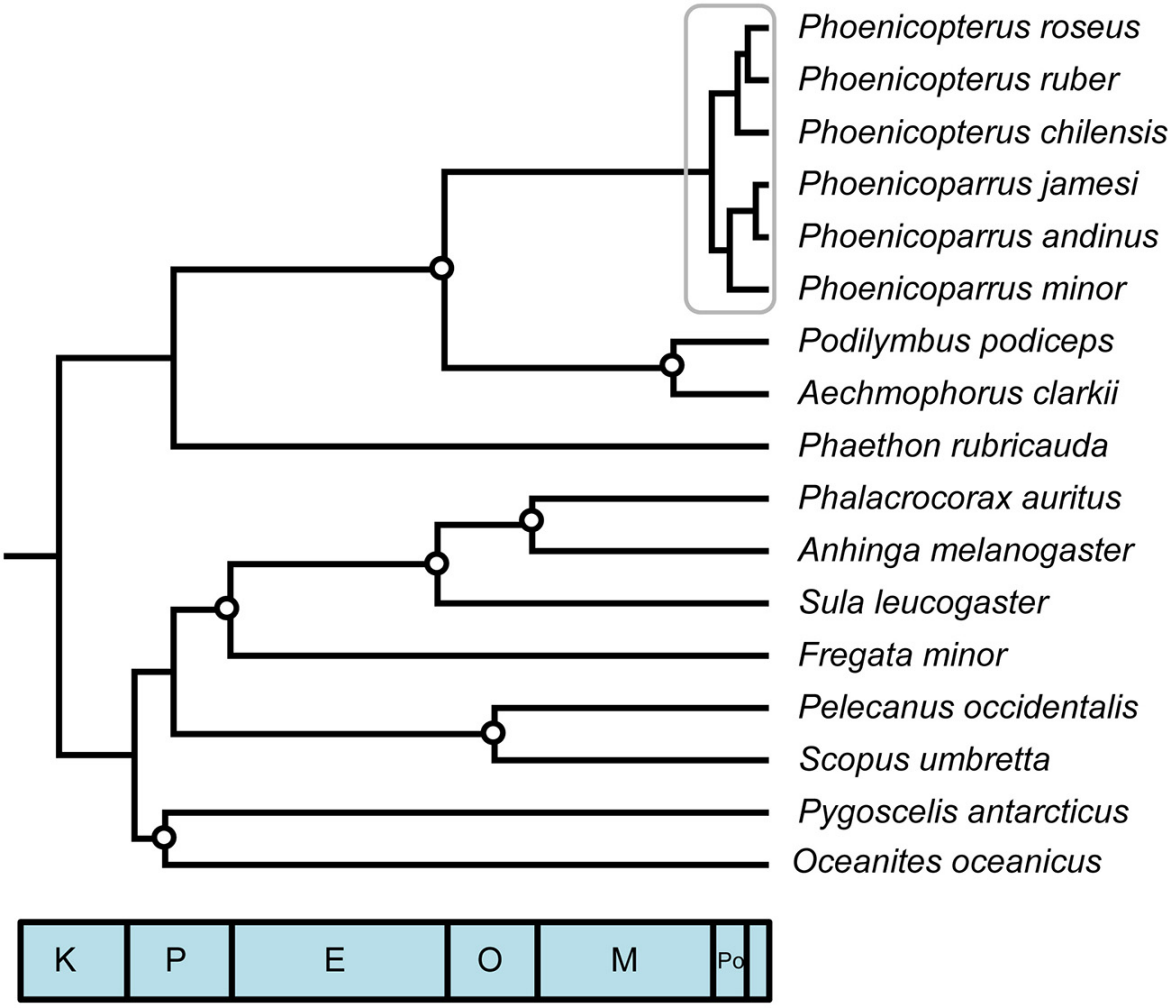
**Phylogenetic reconstruction and reference timetree of aquatic avian ordinal relationships, including complete species sampling of focal young clade, the flamingos (gray box; crown Phoenicopteridae)**. Circles designate fossil constraint placement used in divergence time analysis. See text and **Table 1** for details of reference analysis. All nodes received complete Bayesian support values; all nodes received >99% bootstrap support in ML analyses, except placement of Phaethontidae + Mirandornithes (85%), and Sphenisciformes + Procellariiformes (75%).

A phylogeny constructed from a fully partitioned multi-locus sequence dataset including all six extant flamingo species, closest relatives (Podicipedidae) and representative outgroup taxa from various aquatic families that comprise temporally spaced, fossil constraints (**Figure 1**) highlights several previous findings. Recovered relationships among extant Phoenicopteridae, monophyly of Mirandornithes (=‘Phoenicopterimorphae,’ Jarvis et al., 2014) and monophyly of the waterbird clade Aequornithes that excludes both Phaethontidae and Mirandornithes all reflect recent findings (Van Tuinen et al., 2001; Ericson et al., 2006; Hackett et al., 2008; Jarvis et al., 2014; Torres et al., 2014). These nodes were again recovered when jointly estimating divergence times and phylogeny using the same data set and multiple variants of these data that subsampled taxa, loci, and priors through bootstrapping, jackknifing, or randomization. While phylogenetic relationships within Aequornithes (particularly the relative placement of Sphenisciformes + Procellariiformes to other families) were prone to vary when number of loci was reduced, monophyly was consistently recovered for all fossil-calibrated nodes.

### Divergence Times

Next, divergence time estimates and 95% confidence intervals (CI) were obtained for the nodes in the reconstructed phylogeny using an analysis based on settings that were deemed most appropriate (from here on out referred to as the “reference analysis”). This analysis was based on the most extensive inclusion of taxa (for Phoenicopteridae and representative outgroups), loci and calibrations provided with fossil-based minimum estimates and log-normal distributions, whilst guaranteeing complete sequence coverage for all taxa and loci. Divergence time estimates of nodes on which priors were placed yielded posterior estimates close to the minimum prior estimate. The absolute 95% Bayesian inference CI increased with posterior nodal age, but relative CI (CI range/modal nodal age) decreased from 239% for the youngest node to ∼15% for the oldest nodes. Thus, reporting of this “reference” analysis alone would suggest minor temporal variation in accuracy among priors with increasing precision for older nodes. However, further analyses indicated a far more complex picture.

**FIGURE 2:**
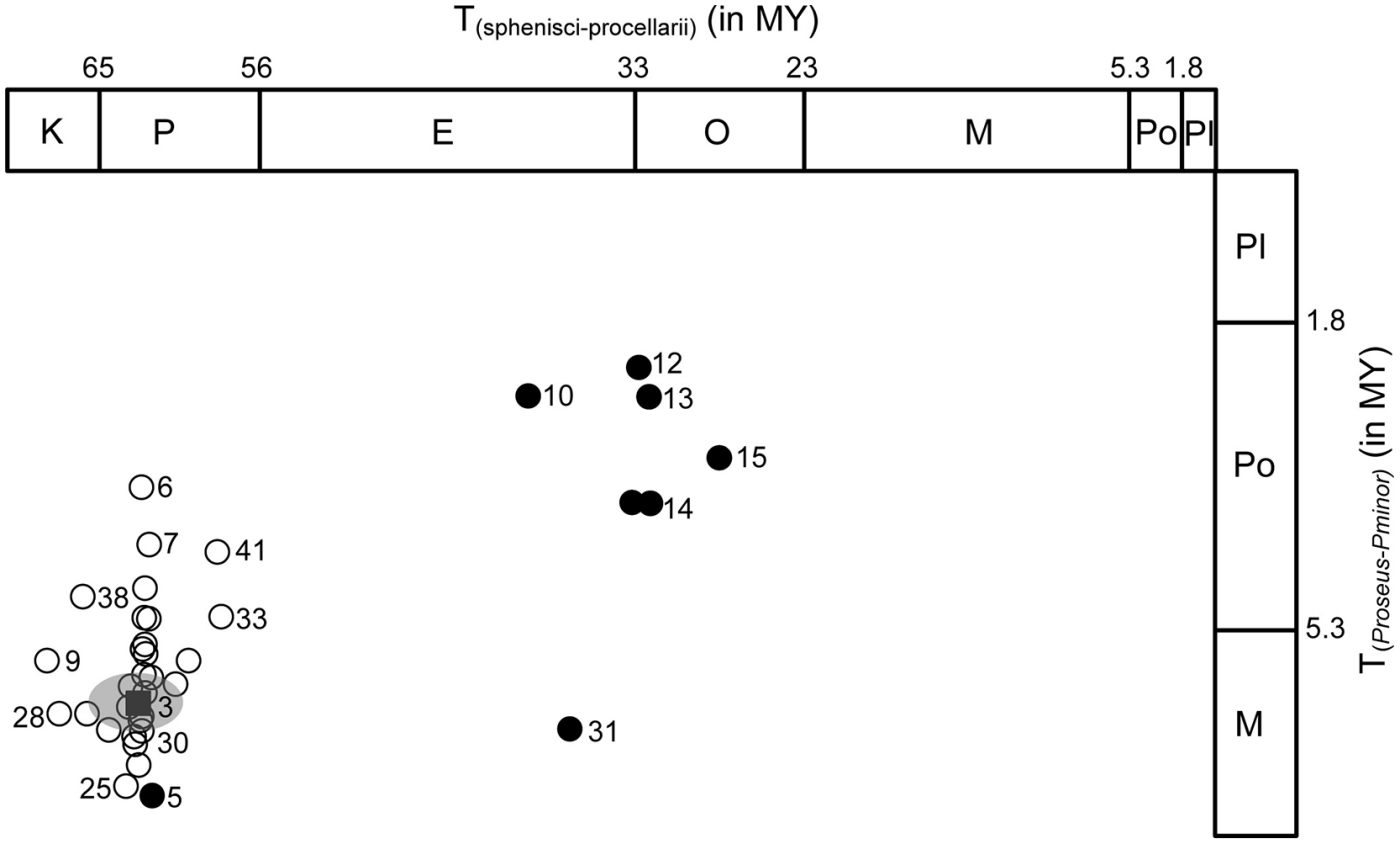
**Divergence time scatterplot of a representative young and old aquatic bird node, representing focal clade Phoenicopteridae (Y-axis) and oldest, Sphenisciformes–Procellariiformes, clade (X-axis)**. Circles show pairwise divergence times from each of 45 sensitivity analyses (see **Table 1**). Age and 95% CI for reference analysis are indicated by black square and gray circle. Closed circles show divergence time results from analysis using youngest constraints only. Numbers next to circle designate analyses listed in **Table 1**.

Evaluation of divergence times across 45 sensitivity analyses (**Table 1**) using different settings related to locus and taxon sampling and priors supplied, yielded significant variation pertaining to nodal age (**Table 2**; **Figure 2**). This variation is documented here on a node-by-node basis in the reconstructed Aequornithes + Mirandornithes phylogeny, with nodes increasing in age from <1 MY (*Phoenicoparrus jamesi*–*Phoenicoparrus andinus*) to approximately 62 MY for the divergence of Procellariiformes–Sphenisciformes. Variation among duplicate runs for a given MCMC analysis is not reported here but in all cases was found to produce relatively minor variation in divergence times (i.e., converged to statistically indistinguishable divergence times and CI intervals).

**Table 2:** Variance in divergence times for a representative young^1^ and old^2^ node in the aquatic bird phylogeny estimated from comparing 45 comparative MCMC divergence time runs to the time estimates of the reference analysis (see text for details).

Parameter	Young^1^	Old^2^	Comment
Taxon sampling	– (42)	NA	2 vs. 6 ingroup (flamingo) taxa
Outgroup sampling	0 (0)	0 (0)	Number of outgroup taxa
Locus sampling	– (30)	0 (1)	4 vs. 5 vs. 6 vs. 7 loci
Genome sampling	+ (208)	0 (1)	mtDNA vs. nDNA
Calibration sampling-1	0 (3)	0 (5)	2 vs. 3 vs. 4 vs. 5 vs. 6 calibrations^3^
Calibration sampling-2	– (58)	– (41)	Young vs. old calibrations
Calibration sampling-3	0 (5)	0 (1)	Narrow vs. broad prior distribution^4^

Numbers between parentheses are maximum percentage deviation in divergence times, estimated from prior time estimate deviating most from reference time estimate/reference time estimate.

^1^*Proseus*–*Pminor* divergence.

^2^Procellariiformes–Sphenisciformes divergence.

^3^Insignificant effect of number of calibrations is found when incorporating at least one old calibration. When this requirement is not met, see calibration sampling-2.

^4^Both uniform and lognormal distributions were tested. Broad lognormal distributions did have a noticeable positive effect (+45% deviation from reference analysis) on divergence time of nodes of intermediate age.

### Nature of Loci (mtDNA vs nDNA)

Analysis of mtDNA alone or in combination with nDNA consistently yielded older ages for the younger nodes (as much as ∼200% when mtDNA was combined with nDNA). However, inclusion of mtDNA appeared to not impact divergence time estimation ages for the oldest nodes, converging to the same nodal ages as reported in the reference analysis.

### Taxon Sampling

We next investigated the effect of ingroup sampling, particularly on the young crown clade Phoenicopteridae. This clade consists of two well-supported genera (Torres et al., 2014), each including three species of flamingos. When comparing divergence time results from analyses with only one representative per genus (*n* = 2 species) to results from analyses with all (*n* = 6) species included, age of this node was consistently underestimated (**Table 2**). No significant effect was uncovered with varying the number of outgroup taxa or when excluding sequences of the flamingo sistergroup Podicipedidae from analysis.

### Locus Sampling

Different combinations of loci were tested for posterior age variation, and indicated variable influence on divergence time estimation. Several combinations of 4, 5, or 6 loci showed significant fluctuation in age of the youngest three nodes, but this pattern was not observed for the deeper nodes where all combinations yielded the same posterior ages (**Table 2**). Furthermore, with increasing numbers of loci the estimated age of the youngest node (*P. andinus–P. jamesi*) in the tree decreased while the estimated age of another young node (crown Phoenicopteridae) increased. Other nodes were constrained by prior information, and the effect of variation among loci did not appear to overwhelm the effect of the joint prior on the constrained nodes, thus yielding identical posterior ages regardless of number and identity of nDNA loci included. When these constraints were removed, an increase in loci affected many of these older nodes by reducing their ages substantially. Because prior constraints appeared to influence our interpretation of variation in nodal ages, we next tested different distributions and combinations of constraints.

### Prior Shape Distribution

Broadening of the prior shape range by increasing the maximum age of stem Mirandornithes, Fregatidae, Sulidae, and Pelecanidae to 65 MY and crown Podicipedidae to 30 MYA under uniform distributions did not influence posterior estimates of these and other nodes (**Table 2**). However, forcing the prior distributions to be lognormally shaped with maxima reaching 65 or 30 MY, significantly increased ages for medium aged nodes, while not impacting the oldest or youngest nodes. Effectively, this approach increases the SD on the lognormal prior distribution to conform to a modal peak significantly older than the minimum age estimate. While alteration of these shapes appears to influence the absolute timing of many nodes, the nodes with the shallowest divergence in our example seem to be spared from this, probably context-specific, bias.

### Calibration Sampling

Considerable variation in estimated divergence times was revealed when altering the combinations of constraints. Use of only the two youngest constraints (crown Podicipedidae; crown Mirandornithes) yielded younger posterior ages for shallow nodes but also significantly underestimated, indeed rejected, nodal ages for which a good fossil record exists (**Table 2**; **Figure 2**): addition of a third constraint recovered ages in agreement with the sulid and phalacrocoracid fossil record, but yielded underestimates still for the oldest fossil records (Fregatidae and Sphenisciformes). In contrast, use of the two youngest constraints in combination with one additional constraint yielded posterior ages for stem Pelecanidae significantly older than its fossil record implies. Thus, with exception of Pelecanidae, the use of up to three constraints significantly underestimated the oldest fossil record of aquatic birds. Adding one more internal calibration (to any node) recovered ages consistent with the fossil record of all groups involved, except for the oldest node. Analyses that used various combinations (in number and age) of constraints revealed that nodal ages across the tree are less impacted by the number of constraints than by the age of the nodal priors. Inclusion of the oldest constraint (open circles in **Figure 2**) with others yielded the oldest nodal ages and was most consistent with the overall fossil record and with results from the reference analysis (black square in **Figure 2**).

### Parameter Convergence

To investigate the effect of overall convergence of the data set on divergence time estimates, we assessed the length of iterations required to reach convergence for the various nodes in the phylogeny, and whether some nodes required longer time to reach convergence. With a standard 10% burnin removed, most of the nodal divergence times quickly reached convergence. The youngest nodes took longest to reach convergence (2–4 M iterations), while the older nodes with supplied priors reached stable divergence times early on in the analyses (0.01–0.1 M iterations). Interestingly, with the exception of the youngest nodes, where suitable ESS values where reached at the same time divergence times stabilized, all divergence times reached a stable state prior to the number of iterations necessary to reach ESS values >100 for the nodal divergence time parameter, but also before ESS values >100 were reached for all calibration parameters. Further investigation on the influence of % burnin and overall length of iterations on reaching stable divergence time estimates indicated that for all nodes with priors supplied <10% burnin and relative few iterations are required; on the other hand, for the nodes where priors were not given, appropriate burnin levels were about 10% with a length of at least 20–40 M iterations run in triplicate to confirm these patterns.

## Discussion

Molecular divergence time estimation inherently carries a degree of uncertainty. Error in molecular clock studies has been linked to substitution rate variation across sites and lineages, substitution saturation, locus sampling, and inaccurate fossil calibration (e.g., van Tuinen and Hadly, 2004). To combat this uncertainty, much focus has been paid lately to a partitioned multi-locus analysis of divergence times under relaxed clock models with calibrated priors supplied on multiple nodes (Ho and Duchene, 2014; Zhu et al., 2014). With this analytical approach, precision of time estimation is enhanced through jointly analyzing many loci (dos Reis et al., 2012; Dos Reis and Yang, 2013; Zhu et al., 2014). Rate variation can be accounted for in specific clock models such as randomized or autocorrelated relaxed clocks (Pereira and Baker, 2006; Brown and van Tuinen, 2012; Ho and Duchene, 2014) or a combination of both (Battistuzzi et al., 2010). Furthermore, accuracy of time estimation is enhanced through inclusion of multiple vetted calibration priors. Because inaccurate fossil constraints determine the principal error in accuracy a best practices approach has been developed to minimize error in accuracy from the vantage point of the fossil record (Parham et al., 2012). Recent reviews (Brown and van Tuinen, 2012; Laurin, 2012; Parham et al., 2012; Ho and Duchene, 2014) and empirical analyses (Marjanović and Laurin, 2007; Inoue et al., 2010; Clarke et al., 2011; Sterli et al., 2013; Warnock et al., 2015) have highlighted the need to incorporate additional paleontological information to better inform the overall prior distribution, particularly the distribution’s shape and maximum age. However, this appeal remains both analytically and logistically challenging to achieve and alternative approaches have been proposed (Pyron, 2011; Wilkinson et al., 2011; Laurin, 2012; Ronquist et al., 2012).

In this study, we investigated to what extent various molecular and calibration sampling schemes differently impact divergence time estimation for topologically shallow nodes compared to deep nodes. We used a multi-locus, multi-calibration approach and focused on flamingos and other aquatic bird families because the divergence time for the common ancestor of extant flamingos is young and the fossil record of flamingos and other aquatic birds provides for several well-vetted constraints. With this “default” approach, we found that the joint use of all available fossil priors yielded temporal consistency with the overall fossil record of aquatic birds and, thus, that there was little evidence for a node age-dependent uncertainty in divergence time estimation. However, through performing additional sensitivity analyses, we uncovered evidence that shallow nodes indeed are differently affected (summarized in **Table 2**).

The evidence for a specific bias affecting shallow nodes covered the variable effect of genomic partitions (mtDNA vs nDNA), taxon sampling, and posterior age convergence that show largest differences in uncertainty between shallowest and deepest nodes. Deviation in posterior ages was largest for the shallowest nodes when comparing between mtDNA and nDNA, incomplete taxon sampling underestimated shallow nodes more than deeper nodes (see Schulte, 2013; Soares and Schrago, 2015 for similar findings), and shallow nodes needed more iterations to converge to a stable evolutionary state (and posterior age) than deeper nodes. However, while this evidence may suggest a node age-dependent uncertainty in divergence time estimation, our additional sensitivity analyses that assessed the effect of different calibration schemes instead indicated the presence of a specific bias through substitutional saturation. Because divergence time bias from molecular homoplasy is also node-age dependent, it can be challenging to ascertain the various factors that account for uncertainty in divergence time estimation.

**FIGURE 3:**
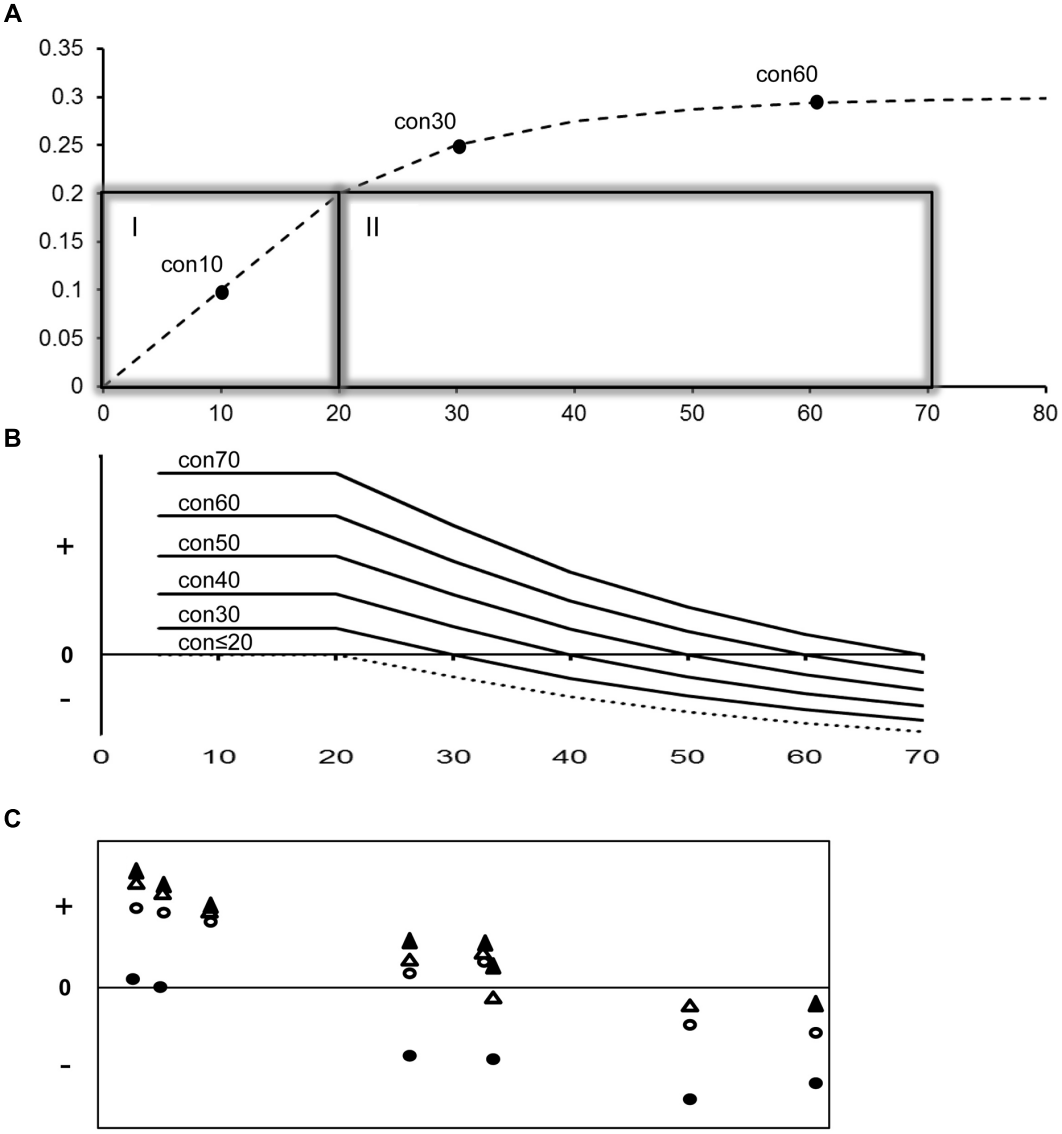
**A model of substitution saturation and the effect of using prior constraints with variable levels of saturation on divergence time**. Output from this model is compared to empirical effects quantified in this study. **(A)** The top panel illustrates a model of substitution saturation with a background rate of 1% per MY that starts showing saturation for sequences diverged more than 20 MY. Divergence time is shown on the X-axis and percent genetic distance on the Y-axis. The presented saturation plot shows a 50% reduction in substitution rate every 10 MY following the 20 MY point, due to increasing homoplasy (back substitutions) with time. Box I and II present the non-saturation and saturation zone. Con 10, 30, and 60 represent three possible fossil constraints with, respectively a 10, 30, and 60 MY minimum age. Note that Con30 and Con60 are both in the saturation zone but at different levels of saturation. **(B)** The middle panel illustrates the variable effect of prior constraint placement on model divergence time estimation with increasing age (X-axis). The Y-axis shows the relative deviation between modeled and estimated divergence times with plus, minus and zero representing overestimation, underestimation and correct estimation of time. Use of young constraints that fall in the non-saturated zone will correctly estimate divergence times up to 20 MY but yield underestimated divergence times for older nodes. Use of older constraints that fall in the beginning of the saturation zone will also yield underestimated divergence time for older nodes, but overestimation of divergence times for younger nodes. The effect of overestimation of younger nodes becomes more pronounced with the age of fossil constraint used. **(C)** The lower panel illustrates the variable effect of prior constraint placement chosen for this study on empirical divergence time estimation with increasing age (X-axis). The X- and Y-axes are scaled the same as in the middle panel. Symbols designate representative constraints of increasing age: crown Podicipedidae (closed circle), stem Sulidae (open circle), stem Fregatidae (closed triangle), and stem Spheniscidae (open triangle). Note the similar trend of overestimation/underestimation with increasing age of constraint and dated divergence (X-axis) between **B** (modeled data) and **C** (empirical data; this study).

Substitution saturation is more extensive in the deeper parts of the tree. Constraining the prior ages of these deeper nodes will thus affect shallow nodes by systematic overestimation and this overestimation increases with (a) level of saturation and, by proxy, substitution rate and (b) temporal depth of nodes (and associated fossils) used as Bayesian constraint. These points are illustrated in **Figure 3** where a known saturation plot is modeled and the effects of nodal depth and differently aged constraints are highlighted. This bias provides an explanation for several of our findings. First, the effect of widening the shape of prior distributions was felt more at deeper nodes. Second, calibration only with young nodes recovered significantly underestimated ages of deeper nodes. Third, posterior nodal ages across the tree were little impacted by the number of constraints, but strongly by the age of the nodal priors, with nearly all posterior ages increasing progressively as posterior minimum ages increase (through addition of priors on older nodes). Four, mtDNA and nDNA regions showed similar posterior ages for the oldest nodes, but deviation in posterior age was progressively larger when the dated nodes are younger. Therefore, even though the simultaneous use of six constraints across the tree may give “appropriate” posteriors on the constrained nodes suggesting effective use of the joint prior, nodes with shallow divergence may be overestimated in this scenario.

Although the use of multiple vetted calibrations should be preferred over the use of single fossil calibrations, bias nonetheless may arise when these constraints are not placed randomly (see Brochu, 2004 for a similar finding based on quartet-dating methods). This bias may appear as a generalized trend of overestimation of divergence times for young nodes because fossil constraints may be placed regularly on deeper nodes yet rarely in the canopy of the tree. Thus, the appropriateness of a given calibration is dependent on the level of molecular saturation and the assignment of calibrations on deeper phylogenetic nodes should not necessarily be favored (Mello and Schrago, 2014) over setting informative priors on shallower phylogenetic nodes.

In a recent review on molecular clock methodology, Ho and Duchene (2014) highlighted the importance of relying on rigorous model selection and accurate informative calibrations, and stated that accurate estimates of timetrees will be aided most by new fossil finds and associated fossil calibration methodology. However, here we surmised that despite choosing informative and vetted fossils and rigorous molecular methods (including use of the most complex substitution models), substitution saturation is not completely accounted for. Saturation is a well-studied phenomenon in mtDNA studies, and can produce divergence time estimation bias in timetree studies (Jansa et al., 2006; Hugall et al., 2007; Lukoschek et al., 2012; Dornburg et al., 2014). Yet, the focus of these studies has been limited to mtDNA. As an alternative to mtDNA, nuclear introns with higher divergence rates are useful for phylogenetic resolution of shallow nodes, but they too can yield biased time estimates of young clades. Substitution variation, including saturation, can be accounted for with complex substitution models and the practice of partitioning across loci. But, it is unclear whether these methods completely correct for multiple substitutions or non-clockiness (Arbogast et al., 2002; van Tuinen and Dyke, 2004; Phillips, 2009; Zheng et al., 2011; Dornburg et al., 2014). Our study thus provides another example of the inability of current models to completely account for substitution variation.

As an alternative to available models, a data set can be recoded to remove excessive variation. The fastest changing substitutions can be removed prior to divergence time estimation, including transitions (RY coding) and third codon positions. For estimation of divergence times of young clades, recoding of the fastest substituting sites may remove saturation but it can also sacrifice phylogenetic information for young clades. In our data set the majority of loci were non-coding and the majority of substitutions providing resolution to shallow clades (e.g., Phoenicopteridae) were transitions.

Because in our case, and likely most cases, the shallowest nodes do not have an easily interpretable fossil record, posterior ages are perhaps inherently more uncertain for younger than older nodes. In this case, the age for *Pjamesi–Pandinus* may be as low as 0.35 MY and as old as 0.65 MY old, both Late Pleistocene ages and not currently testable with the fossil record. Similarly, the age for crown Phoenicopteridae may be as young as 3.2 MY and as old as 5.6 MY, Pliocene or end Miocene ages (**Figure 2**). The fossil record for crown Phoenicopteridae is sparse. Possible crown group flamingos have been reported from as early as late Oligocene (Gervais, 1852). Though some of the earliest of these have been recovered outside of the crown clade (Torres et al., 2015), almost all are known from highly fragmentary material and none can be placed within the crown with any certainty. Consequently, there are no fossil calibrations available for crown group flamingos which meet the criteria proposed by Parham et al. (2012).

Several vetted fossil-based constraints may be included in timetrees focusing on deep topology, yet investigations focusing on young clades will likely not be so fortunate. Our particular example is a lucky one with a well understood and studied stem flamingo and crown grebe fossil record. Most other young systems have limited options for selecting internal fossil priors. None of the textbook adaptive radiations have a suitable recent fossil record, and instead are often dated using either geological calibration (Fleischer et al., 1998; Won et al., 2006) or using a standard, often untested, substitution rate (Shields and Wilson, 1987; Macey et al., 1998; Weir and Schluter, 2008). Darwin’s Finches (Sato et al., 2001; Burns et al., 2014), Hawaiian Honeycreepers (Lerner and Fleischer, 2011), South American *Sporophila* seedeaters (Campagna et al., 2012), and North American *Dendroica* warblers (Lovette and Bermingham, 1999) have all been dated to be <6 MY old, whereas the radiation of African cichlids appears to be slightly older (9–12 MYA; Won et al., 2006). The calibration methods utilized to infer these young ages carry intrinsic assumptions, yet we suspect that the alternative use of external calibrations would also introduce biased divergence times.

In summary, while uncertainty in divergence time estimation is assumed to be less for recently evolved clades than deeper phylogenetic nodes, when young clades are dated with old external priors, the timetree of the Canopy of Life will also carry considerable uncertainty. Given the current explosion of genomic data related to species-level divergence, numerous attempts will soon be made to estimate divergence times from shallow clades. Furthermore, genomic data will be combined to allow for analysis at broader phylogenetic depth, at which both phylogenetic and divergence time estimation will have to accommodate analysis across numerous genomic regions and substitution rates. Thus, in anticipation of these efforts, more investigation is needed to find solutions to estimating divergence times in the canopy of the Tree of Life. One possible solution would be to identify markers that do not show signs of saturation across a given topology.

## Conflict of Interest Statement

The authors declare that the research was conducted in the absence of any commercial or financial relationships that could be construed as a potential conflict of interest.
